# Comparison of measurements of mandible growth using cone beam computed tomography and its synthesized cephalograms

**DOI:** 10.1186/1475-925X-13-133

**Published:** 2014-09-10

**Authors:** Hsien-Shu Lin, Jia-Da Li, Yunn-Jy Chen, Cheng-Chung Lin, Tung-Wu Lu, Mu-Hsiung Chen

**Affiliations:** School of Dentistry, National Taiwan University, Taipei City, Taiwan; Institute of Biomedical Engineering, National Taiwan University, Taipei City, Taiwan; Department of Orthopaedic Surgery, School of Medicine, National Taiwan University, Taipei City, Taiwan; Department of Dentistry, National Taiwan University Hospital, Taipei City, Taiwan

**Keywords:** CBCT, Cephalograms, Mandible growth, Miniature pigs

## Abstract

**Background:**

The current study aimed to compare the measurements of the mandible morphology using 3D cone beam computed tomography (CBCT) images with those using 2D CBCT-synthesized cephalograms; to quantify errors in measurements based on 2D synthesized cephalograms; and to clarify the effects such errors have on the description of the mandibular growth.

**Methods:**

Mandibles of six miniature pigs were scanned monthly using CBCT over 12 months and the data were used to reconstruct the 3D bone models. Five anatomical landmarks were identified on each bone model, and the inter-marker distances and monthly distance changes were calculated and taken as the gold standard. Synthetic 2D cephalograms were also generated for each bone model using a digitally reconstructed radiography (DRR)-generation method. Errors in cephalogram measurements were determined as the differences between the calculated variables in cephalograms and the gold standard. The variations between cephalograms and the gold standard were also compared using paired t-tests.

**Results:**

While the inter-marker distance increases varied among the marker pairs, all marker pairs increased their inter-marker distances gradually every month, reaching 50% of the total annual increases during the fourth and fifth months, and then slowing down in the subsequent months. The 2D measurements significantly underestimated most of the inter-marker distances throughout the monitoring period, in most of the monthly inter-marker distance changes during the first four months, and in the total growth (*p* < 0.05).

**Conclusions:**

Significant errors exist in the measurements using 2D synthesized cephalogram, underestimating the mandibular dimensions and their monthly changes in the early stages of growth, as well as the total annual growth. These results should be considered in dental treatment planning at the beginning of the treatment in order to control more precisely the treatment process and outcome.

## Background

Dental treatments are often performed while the mandible bone is still growing. This is especially true during the process of orthodontic treatment planning or the assessment of the outcome of craniomandibular surgery. Since the data of the growth of the mandible are difficult to obtain via human experiments, physicians have to rely on current images to infer future growth of the mandible in planning treatments and assessing their efficacy. Studies on the influence of factors related to the mandibular growth on the treatment outcome are not yet available. Therefore, including growth-related factors in the treatment plan at the beginning of the treatment in order to control more precisely the treatment process and outcome will be helpful for improving the quality of treatment.

The use of cephalograms for long-term, *in vivo* measurement of human mandible growth would require multiple exposures to radiation over the monitoring period, which is not feasible for ethical reasons. A limited number of *in vivo* studies attempted to image mandible shape changes at limited time instances with unequal intervals but failed to describe long-term growth [1, 2]. Predictive mathematical models may be helpful for resolving the above-mentioned difficulties but, to the best knowledge of the authors, no such mathematical models have been reported. Their experimental validations before their clinical applications present another problem. However, long-term follow-up experiments on animal bone-growth can shed light on the growth of the mandible, and can also be useful in the construction and experimental validation of predictive mathematical models. Therefore, considering the medical ethics, animal experiments are indeed necessary. Many of the previous studies on the mandible have used pigs as animal models because their anatomy, physiology, circulatory system, mastication system and teeth germination are very similar to those of humans [3–5]. This is especially true for mini-pigs in which the size and shape of the jaw, occlusion and bone metabolism rate are similar to those of humans [6–9]. Compared to other pig breeds, mini-pigs are small in body size and relatively easy to manipulate in experiments, making them much more feasible for use as subjects in mandible studies.

**Figure 1:**
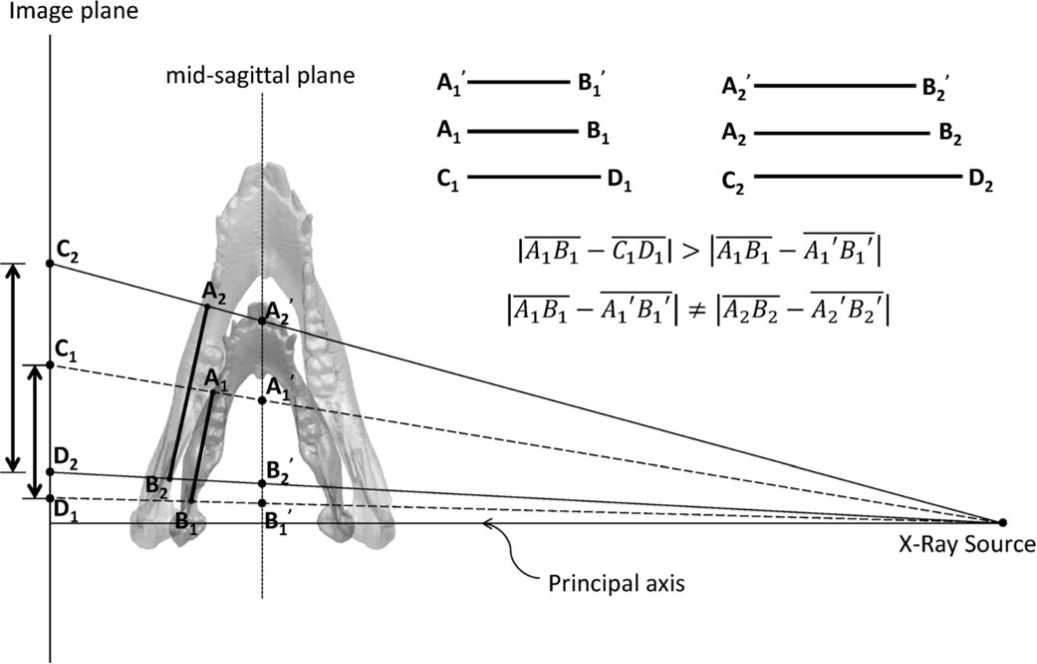
**Schematic representation of two methods.** Schematic diagram showing distances of two bony landmarks measured in the transverse plane from 3D cone-beam computed tomography (CBCT) data (A1, A2, B1 and B2) and from 2D images projected from the 3D CBCT data (Image plane: C1, C2, D1 and D2; mid-sagittal plane: A1’, A2’, B1’ and B2’) at two instances during growth. Differences in the bony landmark positions and the angles between the inter-landmark line segment and image plane lead to different errors in the 2D measurements of the inter-landmark distances, even though the images were scaled to the mid-sagittal plane and the magnification factor was taken into consideration.

Cephalometric analysis is often used as the basis for diagnostic imaging [10–13]. Since it is fundamentally a two-dimensional (2D) projection of the attenuated X-ray through the bones (Figure 1), bones at different distances from the projection plane will produce bone images of different size, position and intensity, leading to errors in measurements [14, 15]. Figure 1 demonstrates the results of a projection of two bony landmarks at two instances during growth. Differences in the bony landmark positions and the angles between the inter-landmark line segment and image plane lead to different errors in the 2D measurements of the inter-landmark distances, even though the images were scaled to the mid-sagittal plane and the magnification factor was taken into consideration. Another source of errors in 2D cephalometry is the errors associated with manual identification of the bony landmarks. Reliability of identification effects on synthesized images has previously been reported [16–18]. Computerized tomography (CT) scans can be used to obtain accurate data of the skull anatomy [19, 20]. Many studies have also shown that the geometric information from the three-dimensional (3D) reconstruction of the skull from the CT data is more accurate than that obtained from 2D images [21–23]. However, no study has evaluated objectively and quantitatively the 2D imaging methods to clarify whether the relevant measurement errors would affect the diagnosis, treatment planning and outcomes. Furthermore, studies are also needed to explore whether such measurement errors using 2D synthesized cephalogram would vary during the bone growth process. Since CT scans are expensive and involve relatively high radiation dosage, they are not suitable for long-term, continuous monitoring. In recent years, cone beam computed tomography (CBCT) has gradually replaced conventional CT in the diagnosis for orthodontic treatment and as an assessment tool in craniofacial surgery [24]. This is mainly because the radiation dose in CBCT is relatively low for small range imaging such as the head and neck [25–27]. However, its radiation dosage is still about ten times higher than that of the cephalogram [28, 29]. Therefore, considering ethical reasons, the cephalogram is still an acceptable solution for bone growth measurement, despite errors associated with X-ray projection and manual identification of landmarks. Therefore, it seems necessary to quantify the errors in 2D measurements based on cephalograms. By taking advantage of the low-dose radiation and accurate 3D craniometry of CBCT, measurement errors in 2D imaging can be obtained by comparing 2D and 3D measurements. This will be helpful for clarifying whether 2D errors would affect diagnosis, and the planning and results of treatment. It will also help clarify whether 2D image-based measurement errors would vary during bone growth.

The purposes of this study were to compare the measurements of mandible morphology using 3D CBCT data with those using CBCT-synthesized 2D images; to quantify the errors in measurements using CBCT-synthesized 2D images; and to clarify whether such errors would affect the description of the changes of mandibular growth.

## Methods

### Subjects and experimental procedure

**Figure 2:**
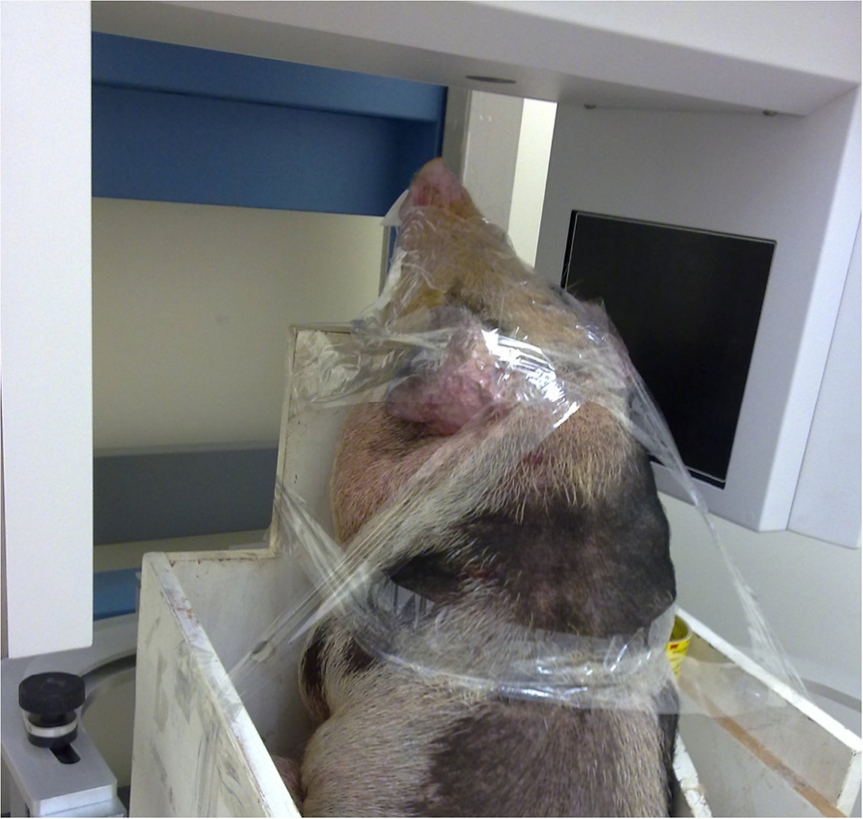
**Experimental setup.** The experimental setup for the CBCT scan of a typical subject. The pig was restrained on a purpose-built workbench using transparent tape, and the mandible was positioned within the center of the region of interest with the guidance of an optical localizer in the shape of a cross.

Six Lee-Sung strain miniature pigs were used in the current study. They were raised on a certified farm for experimental animals (temperature: 26 to 28°C; humidity: 55 to 60%). The pigs were fed normal pig feed, and drank tap water. From the age of one month onwards, each of the pigs underwent a CT scan of the mandible once every month over a period of 12 months. To avoid differences in the number of days between calendar months, and to simplify the description of the changes over the time intervals, a time interval of four weeks was referred to as ‘a month’ (T = time interval = 4 weeks). Therefore, a total of twelve sets of CT data were obtained for each pig (T = T1, T2, …, T12). A low radiation dose CBCT system (i-CAT, Imaging Sciences International, Inc., Hatfield, PA, USA) was used for the CT scanning with an isotropic voxel size of 0.25 mm and a grey scale of 14 bits. The CBCT system was operated with a peak tube potential of 120 kVp and a tube current of 3–8 mA. The field of view was 22 cm (height) × 16 cm (diameter) with the Extended Field of View model provided by the system. A complete scan lasted for 20 s. Before each CT scan, the pigs were administered an intramuscular injection of 1 cc/10 kg of zoletil 50 (50 mg/kg) (Virbac Laboratories, Carros, France) for general anesthesia. Atropine sulfate (Antopin, 1 mg/ml; 0.5 cc: < 20 kg; 1 cc: >20 kg; Sinton Chem & Pharm Co Ltd., Taiwan) was also administered to inhibit saliva production to prevent choking. During the CBCT scan, the pig was restrained on a purpose-built workbench using transparent tape, and the mandible was positioned within the center of the region of interest with the guidance of an optical localizer in the shape of a cross (Figure 2). The study was approved by the Institutional Animal Care and Use Committee.

### Bone model reconstruction and morphological parameters

**Figure 3:**
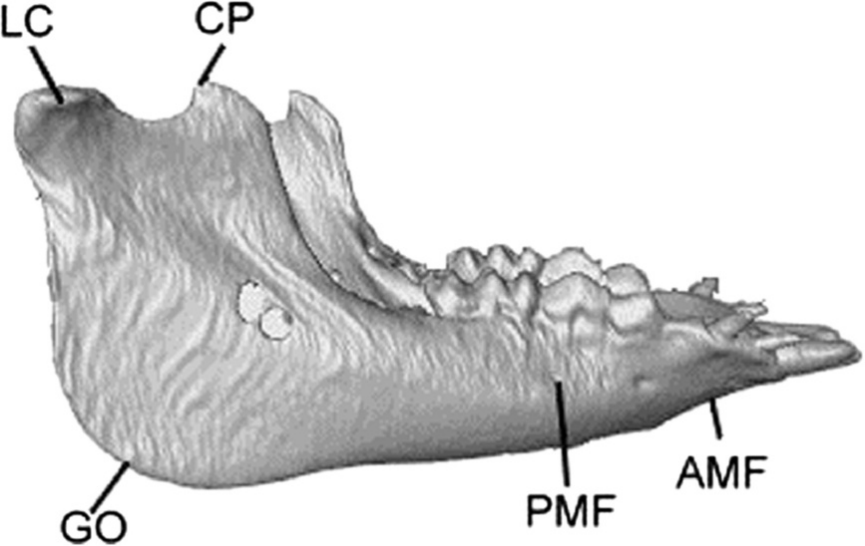
**The chosen anatomical landmarks.** Anatomical landmarks as indicated on the 3D mandible model from the right viewpoint. Definitions of the anatomical landmarks are given in Table 1.

Each of the CT data sets was used to reconstruct a 3D volumetric model of the mandible using Amira (Visage Imaging, Inc., USA) following Lin *et al*. [30]. Selected anatomical landmarks on the mandible were marked on the model to describe the key morphological features of the mandible (Figure 3 and Table 1). These anatomical bony landmarks were selected because they were relatively easy to identify so that the repeatability of identifying the landmarks could be maximized. On each side of the mandible bony landmarks CP, LP and GO were identified automatically by the computer. The CPs were determined as the point of the largest curvature on the coronoid; and the LPs were determined as the most lateral point of the condyle. The GO was determined as the point along the rounded posteroinferior corner of the mandible between the ramus and the body following a geometric approach used by Lin *et al*. [30]. The auto-detected bony landmarks were also verified by an experienced dentist (HSL) before being used for subsequent analysis. Bony landmarks AMF and PMF were identified manually by the same experienced dentist (HSL) within Geomagic 3D Software (Geomagic, Inc., USA) (Figure 3 and Table 1). The reliability of this procedure was determined by the same dentist repeatedly identifying the landmarks, giving an Intra-Class Correlation Coefficient (ICC) of 0.9, which was considered strong for the current purpose.

**Table 1:** **Anatomical landmarks on the mandible utilized in this study**

Anatomical landmark	Definition
LP: lateral pole of condyle	The most protruding point on the lateral side of the mandibular condyle
CP: Coronoid process	The most protruding point on the Coronoid
GO: Gonion	The most posterior and inferior point at the mandibular angle
AMF: Anterior mental foramen	The most anterior edge of the export of the mental nerve
PMF: Posterior mental foramen	The most posterior leading edge export of the mental nerve

### Generation of and measurement on synthesized cephalograms

**Figure 4:**
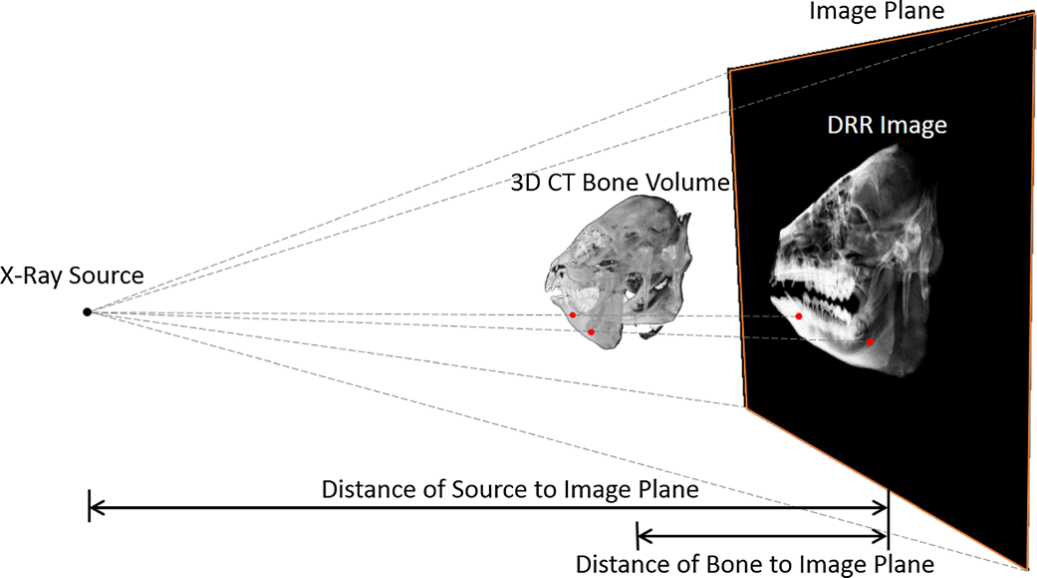
**Simulation of cephalometric imaging.** Simulation of conventional cephalometric imaging using digitally reconstructed radiographs (DRR) from 3D CBCT image data. The DRR of the bones were generated by casting rays from the X-ray source through the volume of the CT-derived bone models. Each of these rays went through a number of voxels of the volume, the attenuation coefficients of which were then integrated along the ray and projected onto the imaging plane to obtain a DRR image resembling a radiograph.

In order to quantify the errors in measurements made on 2D images such as cephalograms as a result of X-ray projection of 3D bone data onto the 2D image plane, the current study used the 3D CBCT data to generate synthesized cephalograms using a digitally reconstructed radiograph (DRR)-generation technique (Figure 4). Given the parameters describing the perspective projection of a cephalogram system, DRR of the mandible can be generated by casting rays from the X-ray source through the volume of the CT data of the mandible. Each of these rays went through a number of voxels of the volume, the attenuation coefficients of which were then integrated along the ray and projected onto the imaging plane to obtain a DRR image resembling a cephalogram [31, 32]. In the current study, projection parameters from a cephalogram system used in the authors’ hospital, namely an Orthoceph OC l00 X-ray system (Instrumentarium Corporation, Imaging Division, Tuusula, Finland), was utilized for simulated projections. The principal axis of the projection was defined as the line connecting the most prominent points on the medial surfaces of the bilateral condyles. The X-ray source was positioned on the right side of the mid-sagittal plane of the mandible at a distance of 1520 mm, whereas the image plane was located 152 mm from the left side of mid-sagittal plane, opposite the source. In other words, the distance from the X-ray source to the image plane was 1.1 times the distance from the X-ray source to the mid-sagittal plane, giving a magnification factor of 1.1. In order to minimize the errors in 2D distance measurements that might result from this magnification, the 2D image was scaled back to the mid-sagittal plane before measurements. In other words, the image was projected to the mid-sagittal plane of the head.

On each DRR-generated synthesized cephalogram, the same anatomical landmarks on the 3D volumetric bone model were also identified by the same experienced dentist (HSL) using a self-developed program (MATLAB, Mathwork Inc., USA). The repeatability of this procedure was determined from the same dentist’s repeated operation, giving a very good intra-rater reliability (ICC > 0.93) [33].

### Data analysis

Since the magnification factor is related to the mid-sagittal plane, bony structures or landmarks away from the mid-sagittal plane will still be subject to various degrees of magnification, and thus errors. These errors cannot be compensated for with a simple scaling factor, and thus were quantified in the current study. In the current study, the measurements made on the 3D CBCT-based model were taken as the gold standard. Therefore, the errors in measurements on the synthesized cephalograms were determined by subtracting the 2D measurements from the 3D gold standard. The 2D cephalogram measurements and their errors during the growth process of the mini-pig mandibles were analyzed using *inter-marker distance*, *inter-marker distance error*, *monthly distance change*, and *monthly distance change error*. The changes in these variables over time represented the changes in the size of the mandible, and the errors associated with synthesized cephalogram measurements. *Inter-marker distance* represented one of the dimensions of the mandible, and was obtained from the coordinates of the identified bony landmarks; *Inter-marker distance error* represented the error in the mandible size measured from a synthetic cephalogram, defined as the difference between the 2D inter-marker distance and the 3D gold standard; *Monthly distance change* represented the amount of monthly growth, defined as the difference in the inter-marker distances between the current and the previous month; *Monthly distance change error* represented the error in the monthly growth of the mandible measured from synthetic cephalograms, defined as the difference in the monthly distance changes between 2D measurements and the 3D gold standard. A summary of the definitions of the variables is given in Table 2. There were ten pairs of markers on both sides of the mandible for inter-marker distance measurements. For describing the growth of the mandible over time, the differences between the inter-marker distances at the 1st and 12th months were also calculated for 2D synthesized cephalograms and the 3D gold standard, giving the total annual increases. The monthly distance changes were also integrated over time to obtain the distance increases as a percentage of the total annual increase. All of the above-mentioned analysis was performed using in-house-developed programs in MATLAB (MathWorks, Inc., USA).

**Table 2:** **Definitions of the variables for the description of the mandibular growth**

Variable	Description
Inter-marker distance	Distance between two bony landmarks measured from CBCT data
Inter-marker distance error	The differences between 3D and 2D inter-marker distance
Monthly distance change	The difference in the inter-marker distances between the current and the previous month
Monthly distance change error	The difference between the 3D and 2D monthly distance change

### Statistical analysis

Apart from descriptive statistical analysis of the calculated variables, paired t-tests were also performed to compare 2D measurements with the 3D gold standard of each of the inter-marker distances, monthly distance changes and total annual increases using SPSS version 13.0 (SPSS Inc., Chicago, IL, USA). A significance level of 0.05 was selected. Considering the resolution of the CBCT (0.25 mm), errors less than 0.25 mm were considered clinically non-significant even if statistical significance (*p* < 0.05) was found.

## Results

**Figure 5:**
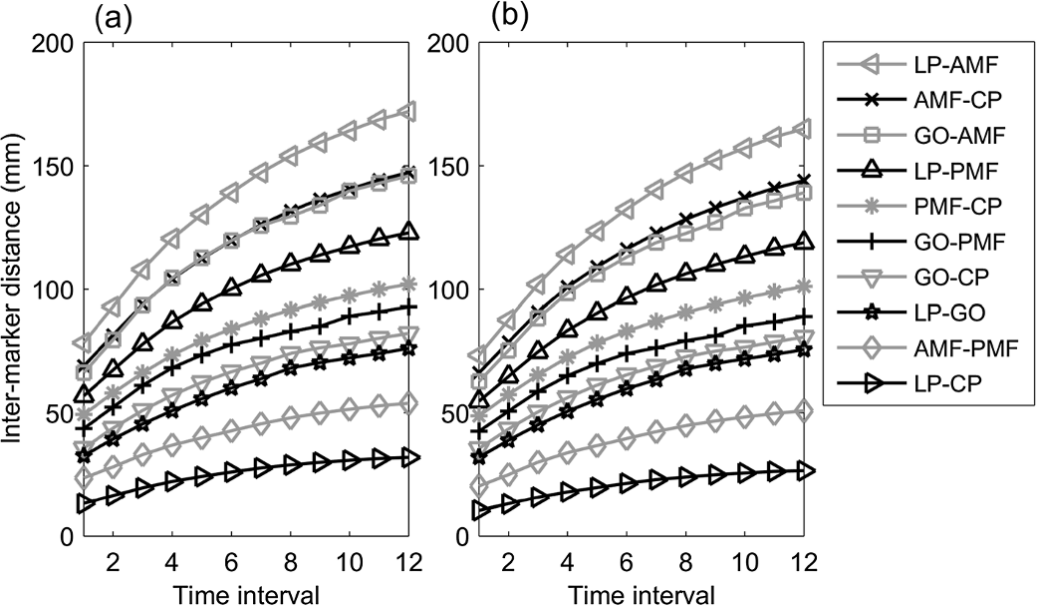
**Mean inter-marker distances over time.** Mean inter-marker distances of the mandible at the 12 time instances of the monitoring period measured from the CBCT data using **(a)** the 3D method and **(b)** the 2D DRR-synthesized cephalograms.

**Figure 6:**
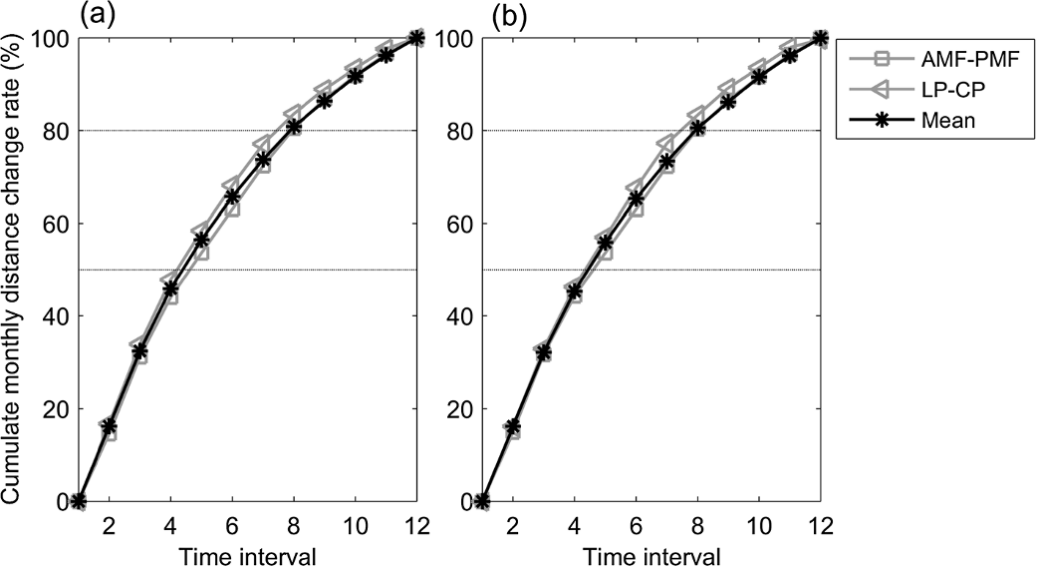
**Change rate of the inter-marker distance over time.** The inter-marker distance increases, calculated by integrating the monthly distance changes over time, as a percentage of the total annual increase, for **(a)** the 3D and **(b)** 2D methods. The mean distance increase of all the marker pairs is shown in blue. The distance increase curves of the fastest increase marker pair (LC-CP) and the slowest marker pair (GO-PMF) are also shown. Note that the distance increase reached 50% of the total annual distance increases during the fourth and fifth months, and reached 80% during the seventh and eighth month.

**Figure 7:**
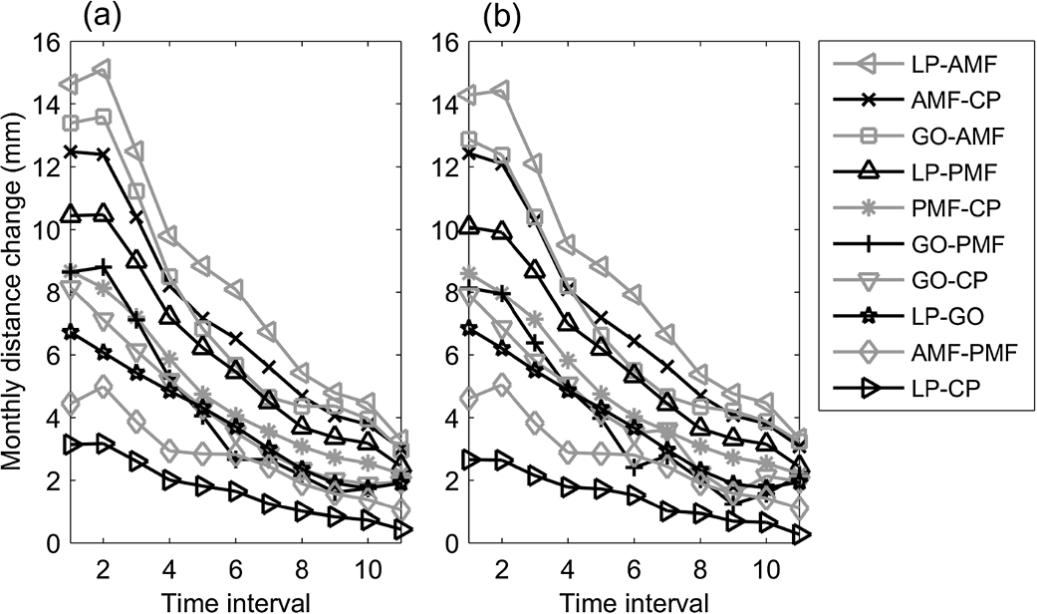
**Monthly inter-marker distance change.** Monthly inter-marker distance change for the mandible over the monitoring period using **(a)** the 3D and **(b)** 2D methods.

The morphological variables calculated from the 2D and 3D measurements were found to be similar in patterns (Figures 5, 6 and 7). The average inter-marker distance increased gradually every month. Among them, the total annual increase for LP-AMF was the largest while that for LP-CP was the smallest (Figure 5). All marker pairs reached 50% of the total annual distance increases during the fourth and fifth months, reached 80% in the seventh and eighth months, and then the increases slowed down during the following four months (Figure 6). This non-linear pattern of growth rate was also revealed by the monthly distance changes over time (Figure 7).

**Figure 8:**
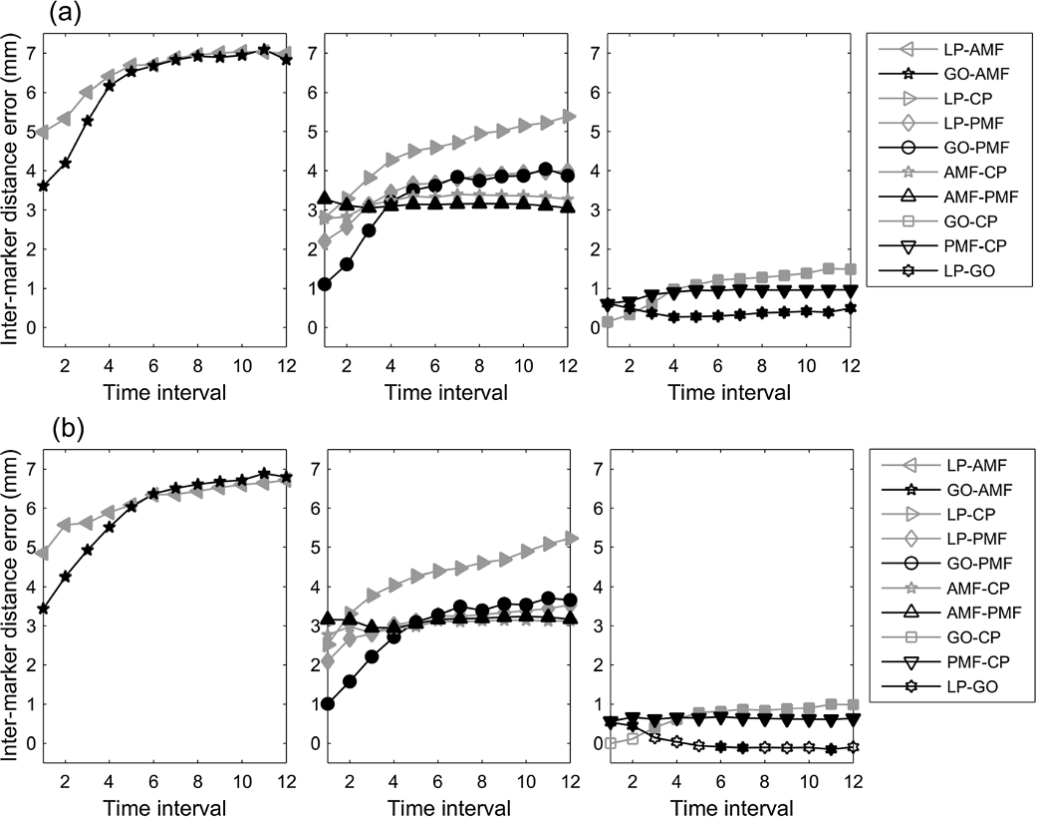
**Mean errors in the inter-marker distances.** Mean errors in the 2D method in measuring the inter-marker distances for the **(a)** left and **(b)** right mandible over the monitoring period. Solid markers on the curves indicate significant differences between data measured using 3D and 2D methods (i.e., p < 0.05).

**Figure 9:**
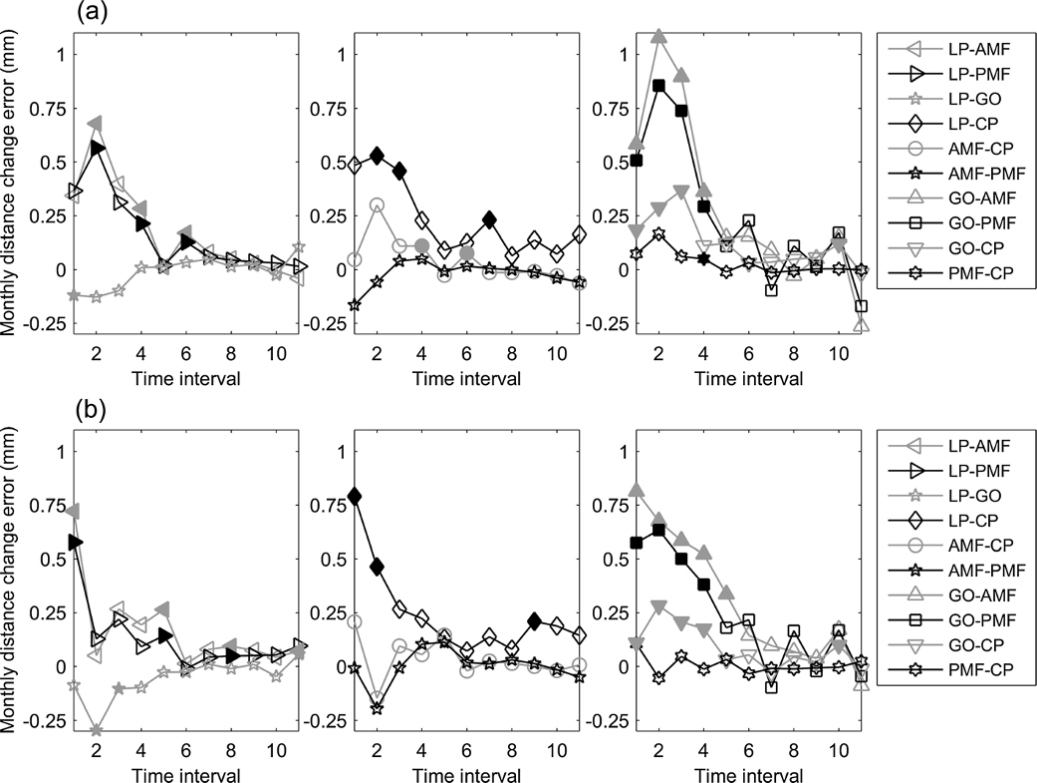
**Mean errors in the monthly distance changes.** Mean errors in the 2D method in measuring the monthly distance changes of the marker pairs for the **(a)** left and **(b)** right mandible over the monitoring period. Solid markers on the curves indicate significant differences between data measured using 3D and 2D methods (i.e., p < 0.05).

The 2D total annual distance increases in LP-AMF, GO-AMF, LP-PMF, GO-PMF, GO-CP and LP-CP were found to be significantly smaller than those determined by 3D measurements (Table 3). The errors in 2D measurements of inter-marker distances were also found to cause significant underestimations in the mean inter-marker distances throughout the monitoring period when compared to 3D measurements, except for GO-CP and LP-GO on the right side (Figure 8). Among these errors, LP-AMF and GO-AMF were the largest, while those for LP-GO, GO-CP and PMF-CP were the smallest. Except for LP-GO, GO-CP and PMF-CP, the errors in the inter-marker distances increased gradually over time (Figure 8).The errors in 2D measurements of monthly distance changes were also found to cause significant underestimations in the mean monthly inter-marker distances of most of the marker pairs during the first four months when compared to 3D measurements (Figure 9). Note that significant underestimations in AMF-CP and PMF-CP were less than the CBCT resolution (0.25 mm).

**Table 3:** **Means (standard deviations) of the total annual increase of the inter-marker distances** ^**†**^

	Left side			Right side		
Description	2D (mm)	3D (mm)	***p***-value	2D (mm)	3D (mm)	***p***-value
LP-AMF	91.66 (2.62)	93.66 (3.19)	0.001*	92.32 (2.66)	94.19 (2.69)	<0.001*
AMF-CP	77.83 (4.74)	78.32 (5.05)	0.059	78.78 (4.13)	79.14 (4.18)	0.062
GO-AMF	76.46 (4.74)	79.68 (4.40)	<0.001*	77.11 (4.36)	80.47 (3.73)	0.001*
LP -PMF	64.20 (2.25)	65.99 (2.66)	0.001*	64.78 (1.11)	66.22 (1.47)	0.001*
PMF-CP	52.44 (4.63)	52.80 (4.77)	0.016*	53.31 (3.54)	53.38 (3.66)	0.556
GO-PMF	46.42 (4.82)	49.19 (4.49)	<0.001*	46.96 (2.62)	49.62 (2.65)	0.001*
GO-CP	45.09 (4.29)	46.44 (3.87)	0.009*	45.83 (4.09)	46.81 (3.78)	0.015*
LP -GO	43.55 (2.42)	43.44 (2.46)	0.230	44.32 (2.21)	43.70 (2.29)	0.006*
AMF-PMF	30.48 (2.42)	30.26 (2.33)	0.368	30.60 (3.31)	30.61 (3.08)	0.934
LP -CP	16.15 (2.71)	18.68 (2.75)	0.001*	15.84 (2.21)	18.49 (2.56)	<0.001*

*P-values for the comparisons between 2D and 3D measurements using paired t-tests are also given. An asterisk indicates a significant difference (p < 0.05).

^**†**^The total annual increase of the inter-marker distances defined as difference between the inter-marker distances at T1 and T12.

## Discussion

This study aimed to compare the measurements of mandible morphology made on 3D CBCT data (gold standard) with those on CBCT-synthesized 2D cephalograms; to quantify the errors in measurements made on CBCT-synthesized cephalograms; and to clarify whether such errors would affect the description of the changes in mandibular growth. While the increases of the inter-marker distances varied among the marker pairs, all marker pairs increased their inter-marker distances gradually every month, reaching 50% of the total annual increases during the fourth and fifth months, and then slowed down in the subsequent months (Figure 6). The errors in 2D measurements caused significant underestimations in most of the inter-marker distances throughout the monitoring period (Figure 8), in most of the monthly inter-marker distance changes during the first four months (Figure 9), and in the total growth (Table 3). These results showed that significant errors were prevalent in the 2D measurements of the dimensions and their monthly changes in the early stage of growth, as well as in the total growth of the mandible.

The measurement errors using 2D imaging are related to the fundamental characteristics of the image formation which is based on the projection of X-rays from a point source through the mandible onto the image plane (Figure 1). With point X-ray projections, the centerline of the imaging source is perpendicular to the image plane. The magnification ratio is dependent upon the ratio of the distance between the source and the imaging plane, and the distance between the source and the object. Thus, reducing the distance between source and object will increase the magnification ratio, and *vice versa*. For a 3D solid object with a complicated geometry and thickness, the projection is the result of non-uniform magnification. These characteristics largely explained the observed 2D measurement errors.The errors in the 2D distance measurements appeared to be affected by the inter-marker distances, and the angles between the inter-marker segments and the image plane (projection angle). For example, since GO-CP and LP-GO were on the ramus that was close to a flat plate and nearly parallel with the projection plane, and since these segments were not the longest ones, they were found to have the smallest errors (<1.5 mm) among all the marker pairs (Figure 8). On the other hand, since LP-CP had the greatest projection angle (>30° for both sides), it had considerable errors (2.3-5.2 mm) even though it was the shortest segment (13–32 mm). Marker pairs with similar distances and projection angles had similar magnitudes of inter-marker distance errors (Figure 8).The large errors in measuring inter-marker segments located between the mental foramen and the outer edge of the ramus could also be explained by the distance and angle factors determined largely by the anatomy. Since the mandibular body resembles a “V”-shape in the transverse plane, each side of the body formed an angle with the image plane, contributing to the errors in the inter-marker segments on the mandibular body. Therefore, being the longest segments on the mandible (length 65–170 mm) with projection angles in the range of 15°-20°, LC-AMF and GO-AMF were found to have the largest measurement errors (3.6-7.1 mm). With similar projection angles, shorter segments on the mandible body in space would thus produce smaller errors, between 1.1 and 5.4 mm (Figure 8).

The error on the side of the mandible further away from the image source was mostly much greater than that on the side closer to the source. In the current study, 2D projection measurements were made on images calibrated back to the median plane. This caused a magnification effect for the side of the mandible closer to the image source but a shrinking effect for the side further away from the source. As indicated in the aforementioned information, the angle formed by the inter-marker segments and the projection plane often led the 2D measurement methods towards a trend of underestimating the true segment length as determined by the 3D methods. Therefore, considering the relative positions of the mandible, image source and image plane, it appears that the magnification effects would compensate for the errors in results for the side closer to the image source while further underestimation of the true length would be expected. It was also observed from the results of the current study that the error of LP-GO on the side of the mandible further away from the image source was greater than that on the side closer to the source, and the difference increased with time.

For many years, the 2D cephalogram has been used by orthodontists as the standard method for assessing the changes of facial bone morphology. Two-dimensional cephalogrametric measurements have also been used to describe the growth of the facial bones. However, the facial bones are three-dimensional objects. Therefore, many details cannot be measured accurately. The superposition of the images of the bones can also lead to measurement errors owing to human misinterpretation. It would be even more difficult to use 2D images for measuring patients who exhibit non-symmetrical or deformed facial morphology. The current study used low radiation CBCT so it was possible to obtain 3D anatomical measurements over the growth process.

The current study was the first in the literature to evaluate the errors associated with 2D measurements over the growth process. This is in contrast to previous studies on the differences in measurements between 2D and 3D image-based methods using a single object at a set growth point in time. While the current study showed that similar bone growth patterns could be obtained using 2D measurements, the measured amount and rate of change of the bone morphology over time were found to be affected by the errors associated with 2D measurements. Two-dimensional measurements caused significant and different underestimations of the inter-marker distances throughout the monitoring period, of the monthly inter-marker distance changes during the first four months and of the total growth. These errors are difficult to eliminate because of their non-homogeneous and non-linear nature. The measurement errors were proportional to the inter-marker distances and their projection angles that were non-homogeneous within the bone and also related to the non-linear growth of the mandible over time [32]. The current results suggest that 2D measurements of the dimensions and their monthly changes during the early stage of growth, as well as the total growth of the mandible, should be interpreted cautiously.

In the current study the amount of growth recorded in the pigs was approximately equivalent to the growth in humans from birth to the age of nine years. The results suggest that the misinterpretation owing to 2D image errors must be taken into account for dental treatment or craniofacial surgery in a growing mandible. Although the current approach cannot be performed on humans due to ethical considerations, in the future, if radiation doses and costs could be reduced, a 3D image-based method could replace the 2D image-based method in providing more accurate measurement parameters. The viewpoint raised in the current study should be included in clinical considerations when drawing up plans for treatment, taking into consideration the effects of growth time-points in interpreting imaging results.

## Conclusions

Significant errors exist in the measurements using 2D imaging methods, underestimating the mandibular dimensions and their monthly changes in the early stages of growth, as well as total annual growth of the mandible. These results should be considered in dental treatment planning at the beginning of the treatment in order to control more precisely the treatment process and outcome.

## References

[1] Krarup S, Darvann TA, Larsen P, Marsh JL, Kreiborg S (2005). Three-dimensional analysis of mandibular growth and tooth eruption. J Anat.

[2] Reynolds M, Reynolds M, Adeeb S, El-Bialy T (2011). 3-d volumetric evaluation of human mandibular growth. Open Biomed Eng J.

[3] Huh K-H, Yi W-J, Jeon I-S, Heo M-S, Lee S-S, Choi S-C, Lee J-I, Lee Y-K (2006). Relationship between two-dimensional and three-dimensional bone architecture in predicting the mechanical strength of the pig mandible. Oral Surg Oral Med Oral Pathol Oral Radiol Endod.

[4] Ström D, Holm S, Clemensson E, Haraldson T, Carlsson GE (1986). Gross anatomy of the mandibular joint and masticatory muscles in the domestic pig (Sus scrofa). Arch Oral Biol.

[5] Langenbach GEJ, Zhang F, Herring SW, Hannam AG (2002). Modelling the masticatory biomechanics of a pig. J Anat.

[6] Obrez A (1996). Mandibular molar teeth and the development of mastication in the miniature pig (Sus scrofa). Acta Anat (Basel).

[7] Koppe T, Rossmann P, Ohkawa Y, Schumacher GH, Nagai H (1997). The course of the mandibular canal in the growing miniature pig. Okajimas Folia Anat Jpn.

[8] Kuboki T, Shinoda M, Orsini MG, Yamashita A (1997). Viscoelastic properties of the pig temporomandibular joint articular soft tissues of the condyle and disc. J Dent Res.

[9] Ide Y, Nakahara T, Nasu M, Matsunaga S, Iwanaga T, Tominaga N, Tamaki Y (2013). Postnatal mandibular cheek tooth development in the miniature pig based on two-dimensional and three-dimensional X-ray analyses. Anat Rec.

[10] Moyers RE, Bookstein FL, Hunter WS, Moyers RE (1988). Analysis of the Craniofacial Skeleton: Cephalometrics. Handbook of Orthodontics.

[11] Athanasiou AE (1997). Orthodontic Cephalometry.

[12] Halazonetis DJ (2005). From 2-dimensional cephalograms to 3-dimensional computed tomography scans. Am J Orthod Dentofac.

[13] Papadopoulos MA, Jannowitz C, Boettcher P, Henke J, Stolla R, Zeilhofer H-F, Kovacs L, Erhardt W, Biemer E, Papadopulos NA (2005). Three-dimensional fetal cephalometry: an evaluation of the reliability of cephalometric measurements based on three-dimensional CT reconstructions and on dry skulls of sheep fetuses. J Cranio-MaxilloFac Surg.

[14] Broadbent BH (1981). A new X-ray technique and its application to orthodontia: the introduction of cephalometric radiography. Angle Orthod.

[15] Adams GL, Gansky SA, Miller AJ, Harrell WE, Hatcher DC (2004). Comparison between traditional 2-dimensional cephalometry and a 3-dimensional approach on human dry skulls. Am J Orthod Dentofac.

[16] Nalçaci R, Öztürk F, Sökücü O (2010). A comparison of two-dimensional radiography and three-dimensional computed tomography in angular cephalometric measurements. Dentomaxillofac Radiol.

[17] Chen M-H, Chang JZ-C, Kok S-H, Chen Y-J, Huang Y-D, Cheng K-Y, Lin C-P (2014). Intraobserver reliability of landmark identification in cone-beam computed tomography-synthesized two-dimensional cephalograms versus conventional cephalometric radiography: a preliminary study. J Dent Sci.

[18] Chien P, Parks E, Eraso F, Hartsfield J, Roberts W, Ofner S (2009). Comparison of reliability in anatomical landmark identification using two-dimensional digital cephalometrics and three-dimensional cone beam computed tomography in vivo. Dentomaxillofac Radiol.

[19] Mah J, Hatcher D (2003). Current status and future needs in craniofacial imaging. Orthod Craniofac Res.

[20] Cavalcanti M, Rocha S, Vannier M (2004). Craniofacial measurements based on 3D-CT volume rendering: implications for clinical applications. Dentomaxillofac Radiol.

[21] Hildebolt CF, Vannier MW, Knapp RH (1990). Validation study of skull three-dimensional computerized tomography measurements. Am J Phys Anthropol.

[22] DeCoster T, Mercer D, Baldwin E (2012). Comparison of the accuracy of X-ray, 2D-CT, 3D-CT, and physical modeling in classification of fractures about the elbow needing operative treatment. UNM Orthopaedic Research Journal.

[23] Cavalcanti MG, Vannier MW (1998). Quantitative analysis of spiral computed tomography for craniofacial clinical applications. Dentomaxillofac Radiol.

[24] Kumar V, Ludlow J, Mol A, Cevidanes L (2007). Comparison of conventional and cone beam CT synthesized cephalograms. Dentomaxillofac Radiol.

[25] Ludlow J, Davies-Ludlow L, Brooks S (2003). Dosimetry of two extraoral direct digital imaging devices: NewTom cone beam CT and orthophos plus DS panoramic unit. Dentomaxillofac Radiol.

[26] Sukovic P (2003). Cone beam computed tomography in craniofacial imaging. Orthod Craniofac Res.

[27] Farman AG, Scarfe WC (2006). Development of imaging selection criteria and procedures should precede cephalometric assessment with cone-beam computed tomography. Am J Orthod Dentofac.

[28] Gibbs SJ (2000). Effective dose equivalent and effective dose: comparison for common projections in oral and maxillofacial radiology. Oral Surg Oral Med Oral Pathol Oral Radiol Endod.

[29] Roberts JA, Drage NA, Davies J, Thomas DW (2009). Effective dose from cone beam CT examinations in dentistry. Brit J Radiol.

[30] Lin H-S, Chen Y-J, Li J-D, Lu T-W, Chang H-H, Hu C-C (2014). Measurement of mandibular growth using cone-beam computed tomography: a miniature pig model study. PLOS ONE.

[31] Siddon RL (1985). Fast calculation of the exact radiological path for a three-dimensional CT array. Med Phys.

[32] Penney GP, Weese J, Little JA, Desmedt P, Hill DLG, Hawkes DJ (1998). A comparison of similarity measures for use in 2-D-3-D medical image registration. IEEE Trans Med Imaging.

[33] Lin H-S, Lu S-L, Chen Y-J, Lu T-W, Huang Y-D: **Test-retest reliability of morphological measurements of the mandible on cone-beam computed tomography-synthesized cephalograms.** *J Dent Sci* in press

